# Supplementary material for: Comparison of Single Target-Controlled Infusion Pump-Delivered Mixed Propofol and Remifentanil with Two Target-Controlled Infusion Pumps-Delivered Propofol and Remifentanil in Patients Undergoing Breast Cancer Surgery—A Prospective Study
Source: Int J Environ Res Public Health. 2023 Jan 23;20(3):2094. doi: 10.3390/ijerph20032094 (PMC9915350; doi:10.3390/ijerph20032094)
Supplement: Supplementary file 1 [file ijerph-20-02094-s001.zip › ijerph-2109889-supplementary.pdf]

Supplementary Table S1. Remifentanil-propofol mixture use in the clinical or laboratory studies.

Study (n)	Type of procedure	The concentration of RP mixture
2004, Berkenbosch et al.[4] (n=15)	Fiberoptic bronchoscopy	R (15-20 μg.ml^{-1}) and P (10 mg.mL^{-1})
2005, Tsui et al.[5] (n=56)	MRI	R (10 μg.ml^{-1}) and P (10 mg.mL^{-1})
2013, Pedersen et al.[6] (n=120)	MRI	R (10 μg.ml^{-1}) and P (10 mg.mL^{-1})
2020, Lai et al.[7] (n=236)	Colonoscopy	R (2.5 μg.ml^{-1}) and P (10 mg.mL^{-1})
2008, Mandel et al.[8] (n=50)	Colonoscopy	R (10 μg.ml^{-1}) and P (10 mg.mL^{-1})
2012, Joo et al.[9] (n=75)	Breast biopsy	R (25 μg.ml^{-1}) and P (10 mg.mL^{-1})
2001, Joo et al.[10] (n=120)	Shock wave lithotripsy	R (10 μg.ml^{-1}) and P (5 mg.mL^{-1})
1999, Fish et al.[11] (n=71)	Daycase urological surgery	R (2.5 μg.ml^{-1}) and P (10 mg.mL^{-1})
2014, Goudra et al.[12] (n=1)	Recurrent radiation therapy	R (5 μg.ml^{-1}) and P (5 mg.mL^{-1})
2012, Kramer et al.[14] (n=37)	Dental extraction	R (5 μg.ml^{-1}) and P (10 mg.mL^{-1})
2012, Mazanikov et al.[15] (n=81)	ERCP	R (20 μg.ml^{-1}) and P (10 mg.mL^{-1})
2005, Brady et al.[16] (n=49)	Ambulatory surgery	R (25 μg.ml^{-1}) and P (5 mg.mL^{-1})
2020, Bagshaw et al.[17] (n=873)	Variety of procedures	R (5 μg.ml^{-1}) and P (10 mg.mL^{-1})
2021, Bakan et al.[18] (n=96)	Thyroidectomy	R (20 or 30 μg.ml^{-1}) and P (10 mg.mL^{-1})
2000, Stewart et al.[20] (n=12)	A laboratory study	R (5 or 50 μg.ml^{-1}) and P (10 mg.mL^{-1})
2020, Henkel et al.[39] (n=3)	A laboratory study	R (10-50 μg.ml^{-1}) and P (10 mg.mL^{-1})
2022, Wylie et al.[40] (n=5)	A laboratory-based study	R (5 μg.ml^{-1}) and P (10 mg.mL^{-1})

MRI: magnetic resonance imaging; ERCP: endoscopic retrograde cholangiopancreatography;
R: remifentanil; P: propofol.
